# Baicalin Inhibits Hypoxia-Induced Pulmonary Artery Smooth Muscle Cell Proliferation via the AKT/HIF-1α/*p27*-Associated Pathway

**DOI:** 10.3390/ijms15058153

**Published:** 2014-05-09

**Authors:** Lin Zhang, Zhichen Pu, Junsong Wang, Zhifeng Zhang, Dongmei Hu, Junjie Wang

**Affiliations:** 1 Department of Respiratory Medicine, the First Affiliated Hospital of Dalian Medical University, No. 222 Zhongshan Road, Dalian 116000, China; E-Mail: linzhangdr2014@163.com; 2 Department of Clinical Medicine, Yijishan Hospital of Wannan Medical College, Wuhu 241001, China; E-Mail: zhichenp@163.com; 3 Department of Oncology, Dalian University Affiliated Xinhua Hospital, No. 156 Wansui Street, Dalian 116000, China; E-Mail: junsongw0111@163.com; 4 Department of Gastroenterology, the First Affiliated Hospital of Dalian Medical University, No. 222 Zhongshan Road, Dalian 116000, China; E-Mail: zhifengzhang1981@163.com; 5 School of Public Health, Dalian Medical University, 9 Western Lvshun South Road, Dalian 116000, China; E-Mail: dongmeih1979@163.com; 6 Department of Cardiology, the First Affiliated Hospital of Dalian Medical University, No. 222 Zhongshan Road, Dalian 116000, China

**Keywords:** baicalin, pulmonary hypertension, pulmonary artery smooth muscle cells, proliferation

## Abstract

Baicalin, a flavonoid compound purified from the dry roots of *Scutellaria baicalensis* Georgi, has been shown to possess various pharmacological actions. Previous studies have revealed that baicalin inhibits the growth of cancer cells through the induction of apoptosis. Pulmonary arterial hypertension (PAH) is a devastating disease characterized by enhanced pulmonary artery smooth muscle cell (PASMCs) proliferation and suppressed apoptosis. However, the potential mechanism of baicalin in the regulation of PASMC proliferation and the prevention of cardiovascular diseases remains unexplored. To test the effects of baicalin on hypoxia, we used rats treated with or without baicalin (100 mg·kg^−1^ each rat) at the beginning of the third week after hypoxia. Hemodynamic and pulmonary pathomorphology data showed that right ventricular systolic pressures (RVSP), the weight of the right ventricle/left ventricle plus septum (RV/LV + S) ratio and the medial width of pulmonary arterioles were much higher in chronic hypoxia. However, baicalin treatment repressed the elevation of RVSP, RV/LV + S and attenuated the pulmonary vascular structure remodeling (PVSR) of pulmonary arterioles induced by chronic hypoxia. Additionally, baicalin (10 and 20 μmol·L^−1^) treatment suppressed the proliferation of PASMCs and attenuated the expression of hypoxia-inducible factor-α (HIF-α) under hypoxia exposure. Meanwhile, baicalin reversed the hypoxia-induced reduction of *p27* and increased AKT/protein kinase B phosphorylation p-AKT both *in vivo* and *in vitro*. These results suggested that baicalin could effectively attenuate PVSR and hypoxic pulmonary hypertension.

## 1. Introduction

Pulmonary arterial hypertension (PAH) is a life-threatening disease characterized by a sustained increase in pulmonary arterial pressure [1]. The pathogenesis of PAH is complex and poorly understood. Pulmonary vascular structure remodeling (PVSR) is a marker of severe and advanced PAH, which is partly due to the proliferation of pulmonary artery smooth muscle cells (PASMCs) [1–3]. Therefore, the inhibition of the abnormal proliferation of PASMCs may open a new therapeutic window in PAH.

Cyclin-dependent kinases (CDKs) and CDK inhibitors regulate the balance between cell proliferation and cell quiescence [4]. *p27*, a CDK inhibitor, inhibits G1 cyclin/CDK complexes and blocks the G1-S transition in the cell cycle [5]. *p27* blocks the cell cycle at the G0/G1 phase, which is a negative regulator of protein kinases, cyclin/CDK [6]. In the normal cell cycle, the G0/G1-phase shows that *p27* is much higher in expression. After mitogenic stimulation, *p27* is rapidly degraded, then allowing the action of CDK2/cyclin E and CDK2/cyclin A to promote cell proliferation [7]. AKT signaling is important for the degradation or downregulation of *p27* and is also crucial in mediating vascular smooth muscle cell (VSMC) proliferation in response to hypoxia exposure [8,9]. Therefore, agents that can regulate the cell cycle processes in VSMCs may have a role in the prevention and treatment of PAH.

Baicalin has been demonstrated to possess multiple pharmacological activities, which is isolated from *Scutellaria baicalensis*, including anti-oxidation, anti-tumor, anti-inflammation and antiproliferation [10–13]. A previous investigation has elucidated that baicalin inhibits platelet derived growth factor BB (PDGF-BB) stimulated vascular smooth muscle cell proliferation through suppressing PDGF receptor β (PDGFRβ)-extracellular signal-regulated kinase 1/2 (ERK1/2) PDGFRβ-ERK1/2 signaling [13]. In mesenteric artery (MA), baicalin produced MA relaxation by activating large-conductance Ca^2+^-activated K^+^ channels via the cyclic nucleotide-dependent protein kinases pathway [14]. Besides, baicalin shows a stronger cardioprotective effect and inhibits the proliferation of cancer cells via the induction of apoptosis [15–18]. Although baicalin has been found to induce apoptosis and inhibit proliferation through multiple pathways. Whether baicalin has an effect on the hypoxia-induced aberrant proliferation of PASMCs in pulmonary arterial remodeling was unknown. Therefore, we investigated the mechanisms of baicalein-induced anti-proliferation in PASMCs on hypoxic pulmonary hypertension (HPH). The present study was designed to investigate the effects and the underlying mechanisms of baicalin on pulmonary vascular remodeling and PASMC proliferation, especially by elucidating its action on the cell cycle and cyclin-dependent kinase inhibitor *p27* pathway *in vitro*. We found that a chronic hypoxia condition resulted in significantly elevated right ventricular systolic pressures (RVSP), increased the right ventricle/left ventricle plus septum (RV/LV + S) ratio and marked media thickening of pulmonary arterioles. Western blotting data showed that hypoxia reduced the expression of *p27* along with the escalation of AKT/protein kinase B phosphorylation (p-AKT). Baicalin treatment reversed the reduction of *p27*, the elevation of p-AKT, accompanied by the attenuation of pulmonary hypertension and PVSR induced by hypoxia. Consistent with the study *in vivo*, experiments *in vitro* also revealed the anti-proliferation effect of baicalin on PASMCs. The novel information partially explained the anti-remodeling property of baicalin on pulmonary artery in hypoxia-induced pulmonary hypertension rats.

## 2. Results and Discussion

### 2.1. Baicalin Attenuates Chronic Hypoxia-Induced Pulmonary Hypertension and Pulmonary Vascular Remodeling

**Figure 1.**
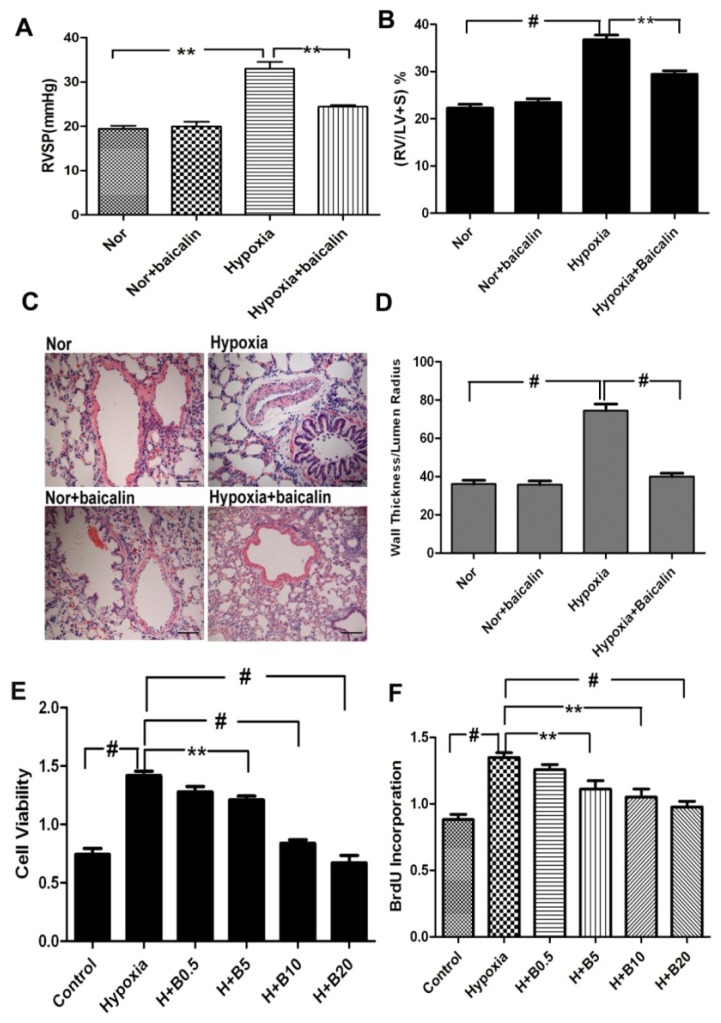
Baicalin attenuates chronic hypoxia-induced pulmonary hypertension and pulmonary vascular remodeling and inhibits rat pulmonary artery smooth muscle cell (PASMC) proliferation under hypoxia exposure. (**A**) Changes in right ventricular systolic pressure (RVSP); (**B**) Changes in the right ventricle/left ventricle plus septum (RV/LV + S) ratio; (**C**) Hematoxylin and eosin staining of pulmonary arterioles (original magnification ×20); (**D**) The ratio of intimal-to-medial areas of the vessel; (**E**) Hypoxia led to a significant increase in cell viability compared with the normoxic condition, while baicalin inhibited the effect in a concentration-dependent manner; (**F**) Hypoxia exposure significantly increased the cell proliferation. However, the hypoxia-induced proliferation of PASMCs was obviously inhibited by various dosages of the baicalin treatment. “Nor” means normoxia; “H” means hypoxia; “B” means baicalin. (^#^ *p* < 0.001; ** *p* < 0.01). All values are denoted as the mean ± SEM from six separate experiments.

As shown in Figure 1A,B, RVSP and the ratio of the weights of the right ventricle to the weight of left ventricle plus septum (RV/LV + S) were much higher in the rat exposed to hypoxia than those exposed to normoxia or the normoxia group treated with baicalin. However, the increase of RVSP and the RV/LV + S ratio was inhibited by the application of baicalin on the hypoxic condition (Figure 1A,B). To evaluate pulmonary vascular remodeling, we examined the medial thickness of the pulmonary arterial walls by hematoxylin and eosin stain (H&E). As shown in Figure 1C,D, hypoxia for four weeks caused significant increases in the thickness of the pulmonary vascular walls in the smooth muscle layer of pulmonary arterioles of the chronic hypoxia group. Hypoxia failed to increase the medial thickness of the pulmonary vascular walls in the smooth muscle layer of pulmonary arterioles in the baicalin treatment. These results indicated that the baicalin treatment prevented hypoxia-induced pulmonary hypertension and pulmonary vascular remodeling.

### 2.2. Baicalin Inhibited Hypoxia-Induced Pulmonary Artery Smooth Muscle Cell (PASMCs) Proliferation

To demonstrate the effect of baicalin on PASMC proliferation, cell viability was determined by measuring 3-(4,5-dimethylthiazol-2-yl)-2,5-diphenyltetrazolium bromide (MTT). We found that hypoxia led to a significant increase in cell viability compared with the normoxic condition. Baicalin inhibited the effect in a dose-dependent manner in the hypoxia condition. At a baicalin concentration of 5 μmol/L, the cell viability of PASMCs was significantly suppressed. Higher concentrations of baicalin (20 μmol/L) almost completely blocked the cell viability induced by hypoxia (Figure 1E). To assess the population of cells, which are actively synthesizing DNA, the 5-bromodeoxyuridine incorporation assay is explored. Our results showed that hypoxia exposure dramatically increased the cell proliferation compared with the normoxia group. The hypoxia-induced proliferation of PASMCs was obviously inhibited by three various dosages of the baicalin treatment (Figure 1F).

### 2.3. Baicalin Effected the Protein Expression of p27 and Hypoxia-Inducible Factor-α (HIF-α) in Rat Lung Tissue and Cultured PASMCs

**Figure 2.**
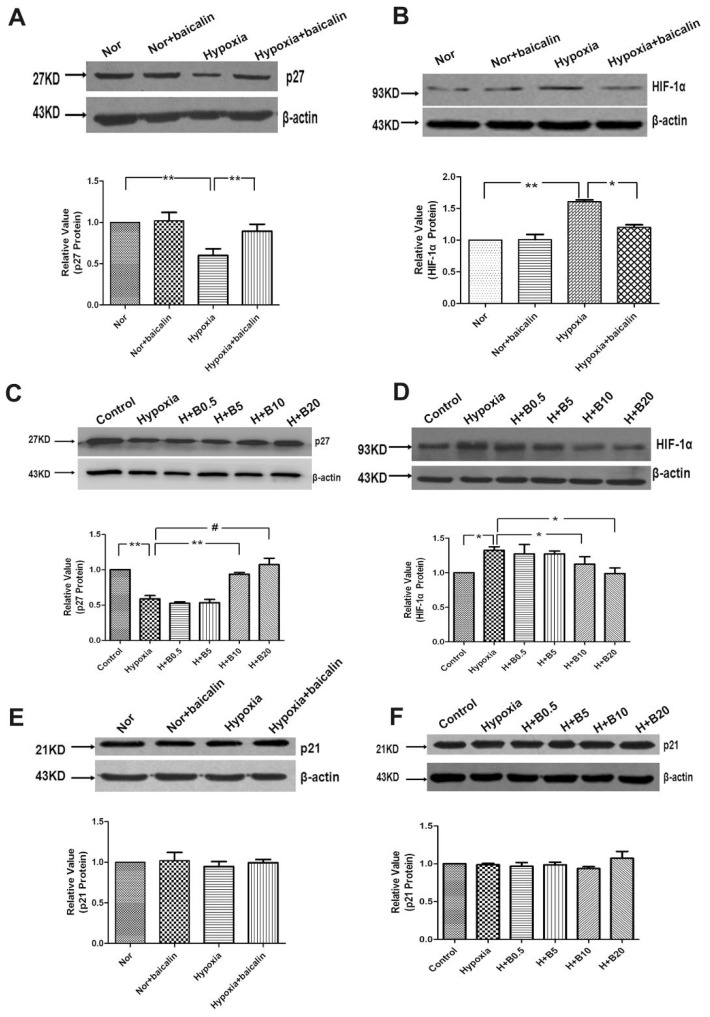
Effects of baicalin on *p27*/*p21* and HIF-1α expression in rat lungs and cultured rat PASMCs. (**A**,**B**) Representative western blotting analysis of *p27* and HIFα protein levels in rat lungs; (**C**,**D**) Representative western blotting analysis of *p27* and HIFα protein levels in cultured rat PASMCs; (**E**,**F**) Western blotting analysis of *p21* protein levels in rat lungs and in cultured rat PASMCs. “Nor” means normoxia; “H” means hypoxia; “B” means baicalin. (^#^ *p* < 0.001; ** *p* < 0.01; * *p* < 0.05). All values are denoted as the mean ± SEM from at least three separate experiments.

Aiming at knowing whether *p27* and HIF-1α were involved in chronic hypoxia-induced pulmonary hypertension and artery remodeling, the protein levels of *p27* and HIF-1α in the four experimental groups were compared (Figure 2A,B). Western blotting results showed that the relative *p27* level in the hypoxia group was significantly lower than that of the normoxia group. The relative *p27* level in the baicalin treatment group was significantly higher than that of the hypoxia group. There was no notable difference of the relative *p27* level between the normoxia and the normoxia + baicalin group. The results also showed that the relative HIF-1α level in the hypoxia group was significantly higher than that of the normoxia group. The relative HIF-1α level in the hypoxia + baicalin group was significantly lower than that of the hypoxia group. In accordance with the results of *p27*, there was no significant change of the HIFα relative level between the normoxia group and the normoxia + baicalin group. To further confirm if *p27* and HIF-1α participated in the process of chronic hypoxia-induced pulmonary artery remodeling, the lysates of each group of PASMCs were used to do western blotting assays. As the results showed, compared with the normoxia group, the relative *p27* level was significant lower in the hypoxia group. However, when PASMCs were treated with various concentrations of baicalin on the hypoxia condition, the expression of *p27* was elevated in a concentration-dependent manner (Figure 2C). The data also revealed that hypoxia exposure notably escalated the expression of HIF-1α compared with the normoxia group. Baicalin treatment resulted in a significant reduction of HIF-1α *vs.* the hypoxia group (Figure 2D). However, the expression of *p21* protein was not markedly altered by the baicalin treatment in hypoxic rat lungs and in cultured rat PASMCs induced by hypoxia (Figure 2E,F).

### 2.4. Protein Expression of p-AKT in Rat Lung Tissue and Cultured PASMCs

**Figure 3.**
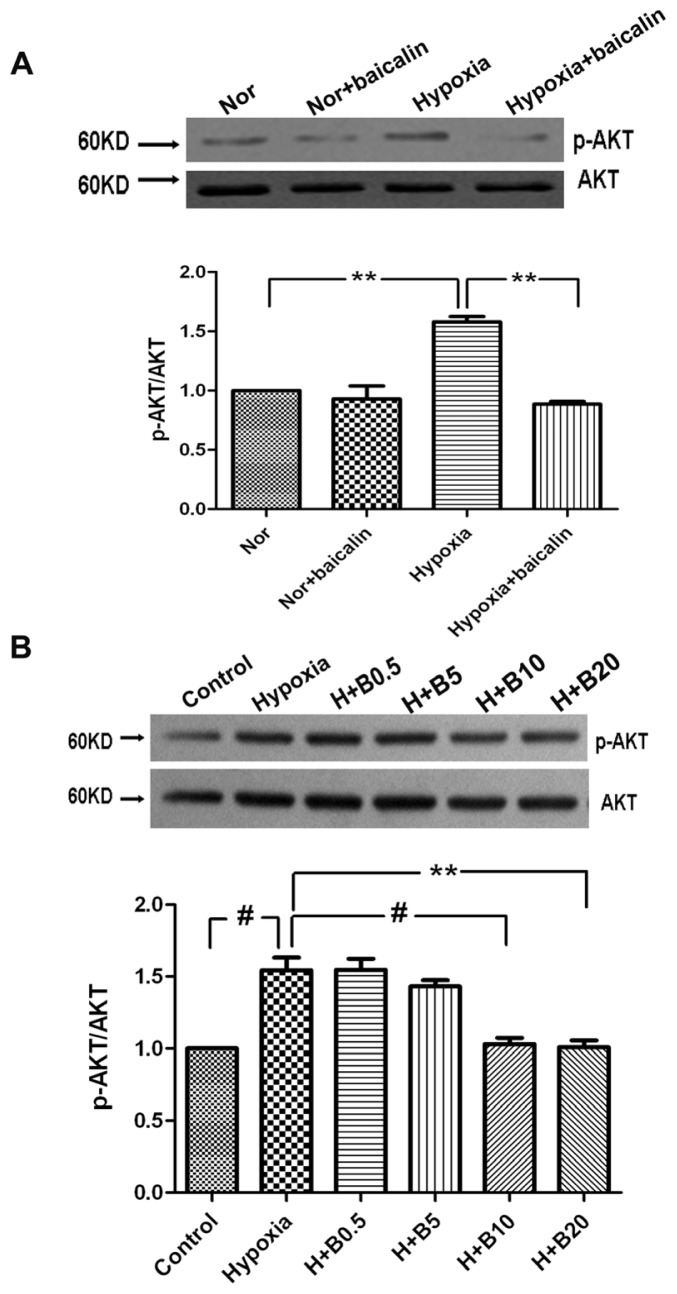
The effects of baicalin on p-AKT expression in rat lungs and cultured rat PASMCs. (**A**) Representative western blotting analysis of p-AKT protein levels in rat lungs; (**B**) Representative western blotting analysis of p-AKT protein levels in cultured rat PASMCs. “Nor” means normoxia; “H” means hypoxia; “B” means baicalin. (^#^ *p* < 0.001; ** *p* < 0.01). All values are denoted as the mean ± SEM from at least three separate experiments.

To further delineate the cellular and molecular mechanisms underlying baicalin-induced PASMCs growth inhibition, we evaluated the effect of baicalin on the PI3K/AKT pathway. Similar to HIF-1α, the relative p-AKT level in the hypoxia group was significantly higher than that of the normoxia group in rat lung tissue. The relative p-AKT level in the hypoxia + baicalin group was significantly lower than that of the hypoxia group (Figure 3A). In accordance with the expression of HIF-1α, in cultured PASMCs, hypoxia exposure resulted in notable elevation of p-AKT *vs.* the normoxia group. The expression of p-AKT in the baicalin treatment groups was lower in a concentration-dependent manner compared with the hypoxia group (Figure 3B).

### 2.5. Changes in mRNA Levels of p27 and HIF-1α in Rat Lung Tissue and Cultured PASMCs

**Figure 4.**
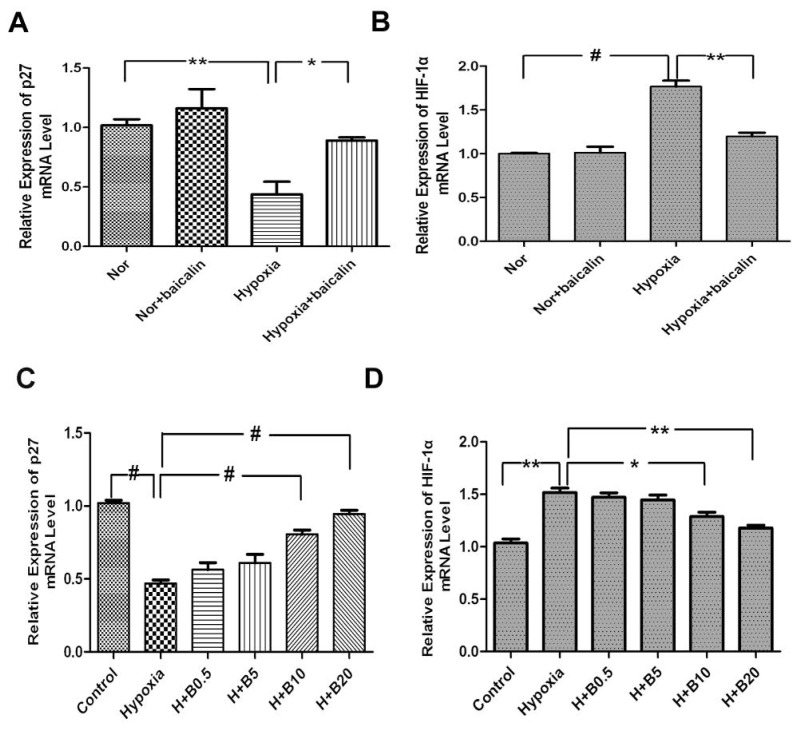
The effects of baicalin on mRNA expression in rat lungs and cultured PASMCs. (**A**,**B**) Analysis of *p27* and HIF-1α mRNA levels in rat lungs; (**C**,**D**) Analysis of *p27* and HIF-1α relative mRNA levels in cultured rat PASMCs. “Nor” means normoxia; “H” means hypoxia; “B” means baicalin. (^#^ *p* < 0.001; ** *p* < 0.01; * *p* < 0.05). All values are denoted as the mean ± SEM from at least three separate experiments.

To further investigate whether *p27* and HIF-1α were regulated at the transcriptional level, the mRNA levels of *p27* and HIF-1α in lung tissue were analyzed by quantitative PCR (qPCR). The results of qPCR showed that there were similar protein levels in all groups whether in rat lung tissue or in cultured PASMCs. The relative *p27* level in the hypoxia group was significant lower than that of the normoxia group. The relative *p27* level in the baicalin treatment group was significantly higher than that of the hypoxia group. There was no notable difference of the relative *p27* level between the normoxia and the normoxia + baicalin group (Figure 4A). Meanwhile, baicalin induced p27 expression in a concentration-dependent manner in PASMCs. Baicalin treatment resulted in significant increase of *p27 vs.* the hypoxia group (Figure 4C). On the other hand, the relative HIF-1α mRNA level in the hypoxia group was significantly elevated compared with that of the normoxia group in rat lung tissue. In the hypoxia + baicalin group, the mRNA level of HIF-1α was significantly reduced compared with the hypoxia group. There was no significant difference between the relative HIF-1α level in rat lung tissue of the normoxia group and the normoxia + baicalin group (Figure 4B). In cultured PASMCs, the relative HIF-1α mRNA level was notably increased after hypoxia exposure compared with the normoxia group (Figure 4D). All different dosages of baicalin treatment in a concentration-dependent manner decreased the HIF-1α mRNA level in cultured PASMCs exposed to hypoxia (Figure 4D).

### 2.6. Discussion

In the present study, we documented that baicalin reversed hypoxia-induced pulmonary artery hypertension. Chronic hypoxia exposure resulted in significantly elevated RVSP, increased RV/LV + S and marked media thickening of pulmonary arterioles. However, the increase was inhibited by the application of baicalin. Further, we showed that, *in vitro*, baicalin downregulated *p27* protein in PASMCs under the hypoxic condition via the HIFα/AKT-associated pathway and that, *in vivo*, baicalin regulated HIF-1α/*p27* in HPH rats. This novel information partially clarified the mechanism of how baicalin ameliorated the hypoxia-induced pulmonary arterial remodeling in HPH rats.

HPH is characterized with a persistent increase in pulmonary artery pressure and pulmonary vascular remodeling, which is a progressive disease with a poor prognosis [19]. Although we have seen advances in the understanding of disease development and treatment, ideal therapies for HPH are still lacking, especially one that can improve the long-term survival of patients with fewer side effects. Baicalin is one of the pharmacologically active components purified from the dried roots of *Scutellaria baicalensis* Georgi, which possesses multiple pharmacological activities, including anti-oxidation, anti-tumor and anti-inflammation [10–12,16]. Previous studies have found that baicalin reduces the protein kinase C (PKC)-mediated MA contractions in endothelium-denuded vessels [20] and produces MA relaxation by activating large-conductance Ca^2+^-activated K^+^ channels via the cyclic nucleotide-dependent protein kinases pathway [14]. Baicalein has demonstrated some anti-proliferative or anti-tumor effects on some cancer cells. Now, baicalin has been widely used in the clinic, especially in China, for the prevention and treatment of cardiovascular diseases and the inhibition of the proliferation of cancer cells via the induction of apoptosis [15–18]. Li *et al*. found that baicalin inhibited PDGF-BB-stimulated vascular smooth muscle cell proliferation [13]. Although baicalin has been found to induce apoptosis and to inhibit proliferation through multiple pathways, the underlying mechanism of baicalin on anti-remodeling in HPH is not well documented. The hypoxia-induced abnormal proliferation of PASMCs is one of the major causes for hypoxic pulmonary arterial remodeling. Therefore, we hypothesized that the inhibition of hypoxia-induced PASMC proliferation may contribute to its anti-remodeling effects in HPH rats. The present study demonstrated that baicalin at 10 and 20 μmol/L significantly inhibited the hypoxia-induced PASMC proliferation without obvious effects on PASMCs under the normoxic condition.

The cell cycle is controlled by cyclin-dependent kinases (CDK) and CDK inhibitors and has been a key therapeutic target in vascular proliferation-associated diseases. *p27*, an important CDK inhibitor, has been found in cancer and vascular diseases. Nabel *et al*. has reported that *p27* is one of the potent inhibitors of vascular smooth muscle cell growth *in vitro* and *in vivo* [21,22]. Fouty *et al*. showed that *p27* modulated PASMC proliferation during mitogenic stimulation, and overexpression of *p27* decreased PASMC proliferation [23]. Consistent with this notion, we have found that hypoxia reduced the level of *p27* protein and mRNA in rat lungs and cultured rat PASMCs. Baicalin reversed the hypoxia-induced reduction of *p27* at protein and mRNA levels. The *p21* gene also plays a key role in the regulation of cell cycle progression, which is another member of the CIP/KIP family of CDK inhibitors. P21 has been reported to have inhibitory effects on pulmonary artery smooth muscle cell proliferation [24], and the role of *p21*/*p53* has been reported to be involved in hypoxia-induced PAH [25]. However, it has been found that *p21* is not necessary for the inhibitory effect of cell growth on hypoxia-induced pulmonary hypertension in mice by heparin [26]. In line with this observation, our data showed that the expression of *p21* protein was not markedly altered by baicalin in rat lungs and in cultured rat PASMCs. It may be that the *p21* signal is not involved in the regulation of hypoxia-induced pulmonary hypertension in baicalin treatment. This finding also indicates different regulation mechanisms of *p27* and *p21*. Hypoxia-inducible factor-α (HIF-α) has been recognized as a master regulatory protein for cells and tissues to adapt to hypoxia. It has been demonstrated that HIF-α is involved in the right ventricular hypertrophy, pulmonary hypertension and pulmonary vascular remodeling. HIF-1α^+/−^ mice showed significantly delayed development of the disease [27,28]. Additionally, adaptation to low hypoxia leads to the transcriptional regulation of multiple genes that participate in cell proliferation and apoptosis [29]. AKT activated HIF-1α protein synthesis and increased HIF-1α protein and transcriptional activity [30]. It has been reported that HIF-1α activity is involved with *p27* expression in cancer cells [31]. Previous studies have reported that AKT is activated in PASMCs in PAH [32]. The activation of AKT was involved in cell growth, proliferation, survival and motility by diverse extracellular signal cascade responses. The activated PI3-K/AKT pathway could enhance *p27* destruction in human cancers [33]. In the present study, we found that baicalin inhibited the hypoxia-induced increase of HIF-1α at both the mRNA and protein levels. Moreover, the hypoxia-induced increase of phosphorylated AKT also was reduced by baicalin. These results suggested that baicalin may protect *p27* against being degraded under hypoxic condition, at least partially, via the AKT/HIF-1α-associated signaling pathway.

It is interesting that *p27* and HIF-1α are involved in the protective effects of baicalin on the chronic hypoxia-exposed rats. In the rat models of the present study, chronic hypoxia exposure resulted in significantly elevated RVSP, increased RV/LV + S and marked media thickening of pulmonary arterioles. Western blotting data showed that hypoxia diminished the expression of *p27* along with the escalation of HIF-1α and p-AKT. Baicalin application reversed the reduction of *p27* and the elevation of HIF-1α and p-AKT, accompanied by the attenuation of pulmonary hypertension and PVSR induced by hypoxia. Consistent with the study *in vivo*, experiments *in vitro* also revealed the anti-proliferation effect of baicalin on PASMCs.

## 3. Experimental Section

### 3.1. Reagents and Antibodies

Baicalin was purchased from Sigma with a purity >99.0%, which was dissolved in dimethyl sulfoxide (DMSO). Antibodies against *p21*, *p27*, HIF-1α and β-actin were purchased from Santa Cruz Biotechnology Inc. (Santa Cruz, CA, USA). Rabbit polyclonal antibodies to AKT and phosphor-AKT were from Cell Signaling Technology, Inc. (Beverly, MA, USA). The bromodeoxyuridine (BrdU) proliferation assay kit was purchased from Roche (Mannheim, Germany). Enhanced chemiluminescence (ECL) reagents were from Amersham International (Amersham, UK). All other reagents were from common commercial sources.

### 3.2. Animals and Lung Tissue Preparation

Adult female/male Wistar rats with a mean weight of 200 g were from the Experimental Animal Center of Dalian Medical University (Grade II), Dalian, China. The animal protocols were approved by the Institutional Animal Care and Use Committee (IACUC). A total of 40 rats were used. Animals were randomly divided into 4 groups: (1) normoxia group (*n* = 6); (2) normoxia group treated with baicalin (100 mg·kg^−1^ via intraperitoneal injection) (*n* = 6); (3) chronic hypoxia group (*n* = 6); (4) chronic hypoxia group treated with baicalin (100 mg·kg^−1^ via intraperitoneal injection) (*n* = 6). The PASMC culture used 16 rats. Rat were treated with baicalin 100 mg·kg^−1^, as previously described [13], and there were some changes on the basis of the previous description. Animals designated for exposure to chronic hypoxia were housed in fractional inspired oxygen at 10% continuing for four weeks. At the beginning of the third week after hypoxia, 6 control rats and 6 hypoxia rats, each rat was given a 100-mg·kg^−1^ baicalin intraperitoneal injection once daily for 14 days. The normoxic control rats were housed in fractional inspired oxygen at 21%. The oxygen concentration (10%) was maintained using a Proox Oxygen Controller (BioSpherix, Lacona, NY, USA). CO_2_ absorption products were used to keep the CO_2_ concentration at less than 0.2%. Anhydrous CaSO_4_ was used to maintain relative humidity within the chamber at less than 60%. The minimal NH_3_ level within the chamber was kept by boric acid. All animals were maintained in a 12:12 h light-dark cycle condition. The room temperature was air-conditioned at 25 °C. At the end of the hypoxia exposure period, we anesthetized each rat with pentobarbital injection (120 mg·kg^−1^, intraperitoneal (i.p.)) and quickly removed the lungs, which were immersed in 4% paraformaldehyde for overnight fixation.

### 3.3. Hemodynamic Experiments

RVSP was measured with a 1.4 F pressure transducer catheter (Millar Instruments, Houston, TX, USA) and AcqKnowledge software (Biopac Systems Inc., Goleta, CA, USA). Briefly, the 1.4 F pressure transducer was inserted through the right external jugular vein of anesthetized rats and threaded into the right ventricle. RVSP was then recorded and analyzed with AcqKnowledge software. After the hemodynamic data were recorded, the thorax was opened. Lungs together with heart were removed to the culture plate with cold PBS. The free wall of the right ventricle (RV), left ventricle (LV) and septum (S) were then carefully dissected and individually weighed to calculate the ratio RV/LV + S as an index of right ventricular hypertrophy. The lungs were immersed in 4% paraformaldehyde for overnight fixation and dissected into 3 mm-thick slices at the same point. The remained lungs were frozen in a −80 °C freezer for subsequent experiments.

### 3.4. Morphological Investigation

The lung tissues were cut from anesthetized rats and embedded in paraffin. The tissues were sectioned into 4 μm-thick sections, and hematoxylin and eosin staining was done.

### 3.5. Cell Culture

The pulmonary arteries were removed from the lungs of adult rats. The outer and inner membranes were removed under anatomy microscope. Minced arteries were digested by 0.2% collagenase type I, then incubated at 37 °C for 1–2 h. The digested pulmonary arteries were centrifuged at 1000 rpm for 5 min and then suspended by Dulbecco’s modified eagle medium (DMEM) containing 20% fetal bovine serum (FBS) and in a 37 °C, 5% CO_2_ humidified incubator. Cells were used between passages 3 and 6. Smooth muscle cell identity was verified by positive staining for smooth muscle α-actin (mouse monoclonal antibody, Sigma, St. Louis, MO USA). Cells in hypoxic conditions were cultured in a Tri-Gas Incubator (Heal Force, Hong Kong, China) with a gas mixture containing 92% N_2_–5% CO_2_–3% O_2_.

### 3.6. 3-(4,5-Dimethylthiazol-2-yl)-2,5-diphenyltetrazolium Bromide (MTT) Assay

Cell viability was analyzed by MTT. PASMCs were cultured in 96-well plates at a density of 1 × 10^4^ cells/well, and then, the cells were treated with different drugs. At the end of the incubation at 37 °C, MTT was added to each well under sterile conditions for 4 h at 37 °C. The supernatant was removed, and dimethyl sulfoxide (200 μL/well) was added to terminate the reaction. The plates were then agitated on a plate shaker. The absorbance of each well was measured at 570 nm in a spectrophotometer.

### 3.7. DNA Bromodeoxyuridine (BrdU) Incorporation Assay

Cell proliferation was assessed using a DNA BrdU incorporation assay (Roche Applied Science, Burgess Hill, UK). PASMCs were seeded into 96-well cell culture plates at a density of 1 × 10^4^ cells/well. BrdU was incorporated into proliferating cells according to the manufacturer’s protocol. Finally, the absorbance of the plate was measured by a spectrophotometer microplate reader at a dual wavelength of 450/550 nm.

### 3.8. Real-Time Reverse Transcription-Quantitative PCR (RT-qPCR)

RNAs were extracted from rat lungs and PASMCs by using TRIzol reagent and then determined by ultraviolet spectrophotometry (absorbance at 260 nm/280 nm). Total RNAs were reverse-transcribed using Superscript First-Strand Synthesis System for real-time reverse transcription-PCR (RT-PCR) according to the manufacturer’s protocol. qPCR was performed with an applied biosystems 7300 fast real-time PCR system. Primers were specifically designed using applied biosystems primer express 3.0 and are listed in Table 1. The specificity of the primers was confirmed with a BLAST program. Each 20-μL reaction contained 1× SYBRR Premix Ex Taq™ II, 10 μM forward and reverse primers, 0.4 μL ROX reference dye and 2 μL of DNA. An ABI 7300 Sequence Detector (PerkinElmer Applied Biosystems, Foster City, CA, USA) was programmed for the PCR conditions: 95 °C for 30 s, 40 cycles of 95 °C for 5 s, and 60 °C for 31 s, followed by routine melting curve analysis. The relative quantitation (RQ) of target gene expression was calculated by the 2^−ΔΔ^*^C^*^t^ method. Each experiment was repeated 2–3 times in 3–4 samples.

### 3.9. Western Blotting Analysis

Pulmonary arteries from rats (normoxia, normoxia with baicalin, hypoxia and hypoxia with baicalin) were homogenized in a hand-held micro-tissue grinder in ice-cold storage protein loading buffer containing protease inhibitors. The homogenates were sonicated on ice and then centrifuged at 14,000× *g* for 10 min at 4 °C. The supernatants were collected and stored at −80 °C until use in western blot analysis.

The cells in 6-well culture clusters were treated with the different experiment group containing 5% FBS/DMEM for 24 h. The cells were lysed in a lysis buffer and incubated for 30 min on ice. Phosphatase inhibitor was added to the lysis buffer. The lysates were then sonicated and centrifuged at 140,000× *g* for 15 min, and the insoluble fraction was discarded. The supernatants were collected and stored at −80 °C until use in western blot analysis. Equivalent amounts of protein (30 μg) from each sample were separated on 12% SDS-polyacrylamide gels and, then, transferred onto nitrocellulose membranes (Millipore, Bedford, UK) and incubated with Tris-buffered saline (Tris 20 mM, NaCl 150 mM, pH 7.6, Tween 20 0.1%) containing 5% nonfat dry milk for 1 h. Membranes were incubated overnight at 4 °C in Tris-buffered saline containing 5% bovine serum albumin and primary antibody, such as anti-*p27* antibody (1:200; Santa Cruz, CA, USA), anti-p-21 antibody (1:500; Santa Cruz, CA, USA) and anti-p-AKT (1:1000; Cell Signaling Technology, Inc., Danvers, MA, USA). Blots were next washed and incubated with an appropriate horseradish peroxidase (HRP)-conjugated secondary antibody in blocking buffer (1:5000) for 1 h at room temperature. Blots were developed using ECL reagent kit (Amersham Biosciences, Little Chalfont, UK).

### 3.10. Statistical Analysis

Data are presented as the means ± SEM. ANOVA and paired or unpaired *t*-tests were performed for statistical analysis as appropriate. *p* < 0.05 was considered statistically significant.

## 4. Conclusions

Collectively, our results demonstrated that baicalin kept *p27* from being degraded through decreasing the production of HIF-1α. Subsequently, *p27* prevented chronic hypoxia-induced PVSR and pulmonary hypertension through its inhibitory effects on PASMCs. Therefore, we concluded that stabilized *p27* by baicalin application indeed participated in the attenuation of PVSR and HPH. The downregulated HIF-α through the AKT signal pathway may be responsible for the increased expression of *p27*. The present study suggested that baicalin, a widely used drug with a well-known safety profile, may serve as a new specific and attractive therapy for HPH.

## Figures and Tables

**Table 1.** Primer sequences used in real-time RT-PCR.

Gene	Primer Sequences(5′-to-3′)	PCR Product Size	Accession Number
*p27*	Forward:5′-CGAACGGGCTCAAGAT-3′ Reverse:5′-CTGTGACGAGGCGATT-3′	198 bp	NM: U10440.1
HIFα	Forward:5′-GAATTAAACCCAAAGAC-3′ Reverse:5′-CAAGAAAGCGACATAG-3′	143 bp	NM: 024359
β-actin	Forward:5′-GTTGACATCCGTAAAGACC-3′ Reverse:5′-GGAGCCAGGGCAGTAA-3′	107 bp	EF: 156276.1
